# Clinical Significance of Credé's Prophylaxis in Germany at Present

**DOI:** 10.1155/S1064744993000080

**Published:** 1993

**Authors:** Udo B. Hoyme

**Affiliations:** 1 Department of Obstetrics and Gynaecology University of Essen Essen Germany; 2 Zentrum für Frauenheilkunde, Universitätsklinikum, Hufelandstrasse 55 Essen D-4300 Germany

## Abstract

The introduction of silver nitrate for prophylaxis of gonococcal ophthalmia neonatorum is one of the milestones of preventive medicine. However, in our time an increasing necessity to review Credé's prophylaxis from both a human rights and a medical standpoint is required. The chairmen of the obstetrics and gynecology departments of the German university hospitals were questioned to learn about their policy and experience. Data were provided by 22 of 28 consulted institutions, representing 31,700 annual deliveries seen over a mean period of 5.5 years. Ocular prophylaxis was in use in 16 (73%) of the reporting hospitals (1% silver nitrate in 14 and gentamicin in 2). A nonspecific conjunctival reaction occurred in 5–10% of the newborns, but no major side effects were seen. Non-gonococcal ophthalmia neonatorum was observed in less than 0.1%; however, institutions without a preventive policy reported up to a 5% incidence of neonatal conjunctivitis, mostly due to *Staphylococcus aureus*, as well as *Neisseria gonorrhoeae* in two newborns. Application of silver nitrate is considered a necessary prophylactic measure and safe if it is properly administered. However, major efforts should be directed toward its replacement by alternative antiseptic substances as well as toward chlamydial screening and therapy in pregnancy.


Infectious Diseases in Obstetrics and Gynecology 1:32–36 (1993)
© 1993 Wiley-Liss, Inc.

### Clinical Significance of Credé's Prophylaxis in Germany at Present

Udo B. Hoyme
*Department of Obstetrics and Gynaecology, University of Essen, Essen, Germany*

**Abstract**

**The introduction of silver nitrate for prophylaxis of gonocöccal ophthalmia neonatorum is one of the milestones of preventive medicine. However, in our time an increasing necessity to review Credé's prophylaxis from both a human rights and a medical standpoint is required. The chairmen of the obstetrics and gynecology departments of the German university hospitals were questioned to learn about their policy and experience. Data were provided by 22 of 28 consulted institutions, representing 31,700 annual deliveries seen over a mean period of 5.5 years. Ocular prophylaxis was in use in 16 (73%) of the reporting hospitals (1% silver nitrate in 14 and gentamicin in 2). A nonspecific conjunctival reaction occurred in 5–10% of the newborns, but no major side effects were seen. Non-gonococcal ophthalmia neonatorum was observed in less than 0.1%; however, institutions without a preventive policy reported up to a 5% incidence of neonatal conjunctivitis, mostly due to *Staphylococcus aureus*, as well as *Neisseria gonorrhoeae* in two newborns. Application of silver nitrate is considered a necessary prophylactic measure and safe if it is properly administered. However, major efforts should be directed toward its replacement by alternative antiseptic substances as well as toward chlamydial screening and therapy in pregnancy.** © 1993 Wiley-Liss, Inc.

Key words
Gonoblennorrhea in newborns, silver nitrate, treatment of chlamydial infection in pregnancy

The establishment of silver nitrate prophylaxis of gonococcal ophthalmia neonatorum is one of the milestones of preventive medicine in the 19th century. In 1881 Carl Siegmund Franz Credé, one of the first editors and founder of the famous journal *Archiv für Gynäkologie,* reported from Leipzig a decrease in the incidence of gonococcal ophthalmia from 7.6% to 0.5% (Table 1) by the use of 2% silver nitrate.[1] In the following years prophylaxis was established and required by law or by health department regulations in many countries all over the world. In Germany enforcement was terminated in 1986 by the proclamation of a new law (Gesetz über den Beruf der Hebamme und des Entbindungspflegers vom 4.6.1986). As a result, an increasing necessity arose to review Credé's prophylaxis from both a human rights and a medical standpoint in the medical profession as well as in the government.

This paper reports on recent prevention efforts in German university hospitals on the basis of a questionnaire. In addition, the literature is evaluated in an attempt to define the current standard of care according to the position of the German Federal Health Department (Bundesgesundheitsamt) at Berlin.

### SUBJECTS AND METHODS

In January 1992, 28 chairmen of the departments of obstetrics and gynecology at the university hospitals in the former West Germany were sent the following questionnaire concerning prevention of ophthalmia neonatorum by Credé's procedure in their hospitals:

Address correspondence/reprint requests to Dr. Udo B. Hoyme, Zentrum für Frauenheilkunde, Universitätsklinikum, Hufelandstrasse 55, D-4300 Essen, Germany.

Received December 8, 1992
Accepted December 8, 1992

Clinical Study

**TABLE 1. Incidence of ophthalmia neonatorum in Credé's original series before and after initiation of prophylaxis with 2% silver nitrate**

| Year | Deliveries (no.) | Ophthalmia neonatorum (no.) | (%) |
|---|---|---|---|
| 1874 | 323 | 45 | 13.6 |
| 1875 | 287 | 37 | 12.9 |
| 1876 | 367 | 29 | 9.1 |
| 1877 | 360 | 30 | 8.3 |
| 1878 | 353 | 35 | 9.8 |
| 1879 | 389 | 36 | 9.2 |
| 1880 (to 5/31) | 187 | 14 | 7.6 |
| 1880 (6/1–12/8) | 200 | 1[a] | 0.5 |

[a]Prophylaxis was not administered in this infant.

1. Do you perform neonatal ocular prophylaxis at your institution? If yes, by what method?
2. Did you observe significant or continuous adverse reactions following this prophylaxis?
3. Are you aware of any case of gonococcal conjunctivitis or of conjunctivitis of other etiology at your hospital?
4. How many deliveries and how many years do the figures include?
5. Do you agree to publication of the information provided concerning your hospital or do you prefer anonymity?
6. Do you want to be informed about the results and the progress of this inquiry?

The last question refers in part to a similar inquiry in the university and other major hospitals in the former East Germany. It was initiated by B. Viehweg at the same time and presented in April 1992.

All data returned are analyzed and presented here, in some cases after a precise check-back with the providing institution. The data obtained were also taken into consideration in the decision-making process of the German Federal Health Department.

### RESULTS

The data requested were provided by 22 of the 28 chairmen between January and March 1992. However, since three of them prefered anonymity, the participating institutions are listed by numbers (Table 2). The results reported represent a total of 31,700 annual deliveries seen over a mean period of 5.5 years (Table 3).

Neonatal ocular prophylaxis was provided in 16 of 22 hospitals (73%). The use of 1% silver nitrate was mandatory in 12 hospitals. Occasional application because of certain indications or at parents' requests was reported by one institution each. Two hospitals performed prophylaxis with gentamicin, one on request and one as a routine procedure.

Ophthalmia neonatorum but no gonococcal disease was observed by those hospitals with prophylaxis in less than 0.1%. On the other hand, institutions without routine preventive policy or with no prophylaxis reported up to 5% prevalence of neonatal conjunctivitis in general as well as two newborns with *Neisseria gonorrhoeae. Staphylococcus aureus* was specified as the major etiologic pathogen by some reporters, and in spite of all diagnostic efforts in the past, chlamydial infection was also demonstrated at four locations irrespective of prophylaxis policy.

Nonspecific conjunctival reaction was reported by 6 of those 12 institutions using 1% silver nitrate routinely, with an estimated peak incidence of 5–10% on day 2. The other six hospitals did not observe this kind of reaction; however, it was also reported by one using gentamicin. Major, long-lasting, or permanent side effects were denied by all corresponding institutions, but discontinuation of prophylaxis was justified by one of them because of the severity of transient reactions. According to Viehwegs inquiry in the former East Germany, silver nitrate was used routinely in 19 of 23 responding hospitals with no reported major side effects or long-lasting damage in 860,300 cases.

### DISCUSSION

Ophthalmia neonatorum is defined as the occurrence of conjunctivitis within the first month of life. *N. gonorrhoeae* causes the most destructive form,[1–5] but other bacterial agents including *Chlamydia trachomatis*, *Staphylococcus aureus*, *Streptococcus* ssp., *Haemophilus* spp., and *Pseudomonas* spp. can also cause neonatal conjunctivitis[3,4,6–9] (Table 4). In general, the incidence of severe ophthalmia nenatorum parallels that of maternal gonorrhea and chlamydial infection; however, other pathogens may also lead to conjunctivitis, with resulting septicemia, nasolacrimal duct obstruction, ulceration, scarring, and blindness in the newborn.[4] This is in part due to the lack of sufficient

TABLE 2. Prophylaxis of ophthalmia neonatorum at German university hospitals: Results of an inquiry in January 1992 [response rate 79% (22/28)]

| Institution no. | Method | Side effects | Ophthalmia | Deliveries/ year | Observation period (years) |
|---|---|---|---|---|---|
| 1 | Credé | None | None | 800 | 12 |
| 2 | Credé | 5% | None | 1,500 | 10 |
| 3 | Credé | Frequent, at day 2 | 0.1% *C. trachomatis* | ? | ? |
| 4 | Credé | None | None | 1,000 | 1 |
| 5 | Credé | None | None | 1,800 | 5 |
| 6 | Credé | 10% | *C. trachomatis* ? | 800 | 5 |
| 7 | Credé | Frequent | 0.1% *S. aureus* | 1,200 | 5 |
| 8 | Credé | None | None | 1,600 | 3 |
| 9 | Credé | None | None | 1,000 | ? |
| 10 | Credé | None | None | 2,000 | 11 |
| 11 | Credé | Unspecific | Unspecific[a] | 1,100 | 13 |
| 12 | Credé | Unspecific | None | 500 | 5 |
| 13 | Credé[b] | None | 1 × *C. trachomatis*, some *S. aureus* | 1,000 | 2 |
| 14 | Credé[c] | None | None | 1,600 | 4 |
| 15 | Gentamicin | Unspecific | 0.05%[a] | 1,800 | 1 |
| 16 | Gentamicin[c] | None | Rare[a] | 1,900 | 1 |
| 17 | None | (Unspecific)[d] | 2 × *N. gonorrhoeae*, others | 2,400 | 10 |
| 18 | None | — | 3–5% *S. aureus* | 1,600 | 5 |
| 19 | None | (Significant)[d] | None | 1,700 | 8 |
| 20 | None | (Unspecific)[d] | None | 1,600 | 1 |
| 21 | None | (Unspecific)[d] | Some *C. trachomatis*, *S. aureus* | 1,000 | 3 |
| 22 | None | (Unspecific)[d] | Unspecific | 2,400 | 6 |

[a]No gonococcal infection.
[b]Prophylaxis administered occasionally.
[c]Prophylaxis on parents request.
[d]Before prophylaxis was abandoned.

amounts of lysozyme, IgA, and lacrimal fluid on the newborn cornea.[3]

First introduced as prophylaxis against gonococcal ophthalmia neonatorum by Carl Siegmund Franz Credé in 1880, silver nitrate remained the standard of care in many countries worldwide; it is or has been required or recommended by state laws or health department regulations or officials.[12,13] In addition, it is listed by the World Health Organization as one of the essential drugs.[14] The current discussion focusses on some of the problems with silver nitrate[12,15]: 1) it does not provide perfect protection against neonatal (gonococcal) ophthalmia; 2) it often causes chemical irritation of the conjunctiva; and 3) its use may be superfluous in some populations. To consider these issues a committee of the German Federal Health Department evaluated the recent situation in Germany and reviewed the arguments for and against prophylaxis of ophthalmia neonatorum in order to establish a recommendation for the standard of care in Germany. The arguments will be discussed here.

TABLE 3. Prophylaxis of ophthalmia neonatorum at German university hospitals: summary of an inquiry in January 1992 [response rate 79% (22/28)]

| | |
|---|---|
| Deliveries/year | |
| Number | 31,700 |
| Range | 500–2,400/hospital |
| Observation period (years) | |
| Mean | 5.5 |
| Range | 1–13 |
| Any prophylaxis | 16/22 (73%) |
| 1% Silver nitrate | 12 |
| 1% Silver nitrate occasionally | 1 |
| 1% Silver nitrate on request | 1 |
| Gentamicin | 1 |
| Gentamicin on request | 1 |
| Prophylaxis | 0–10% Unspecific reactions, occasionally *S. aureus*, 0.1% *C. trachomatis*, no reported gonococcal conjunctivitis |
| "Controls" | 0–5% *S. aureus*, 2 cases of gonococcal conjunctivitis, some *C. trachomatis*, others |

**TABLE 4. Causes of ophthalmia neonatorum in the United States[a]**

| Source | Approximate frequency (%) | Time of onset (days) |
|---|---|---|
| Chemical conjunctivitis | 7 | 6–24 hours |
| *Chlamydia trachomatis* | 3–13 | 5–14 |
| *Escherichia coli* | 5–9 | 5–21 |
| *Haemophilus* spp. | 5 | 5–21 |
| Herpes simplex virus | Rare | 2–14 |
| *Neisseria gonorrhoeae* | 3 | 2–5 |
| *Staphylococcus aureus* | 5–30 | 5–21 |
| *Streptococcus viridans* | 4–16 | 5–21 |

[a]For data, see Dinsmoor[4].

The incidence of *N. gonorrhoeae* in pregnant women in Germany is estimated to be as low as 0.1%, or approximately 700 cases/year. On the other hand, neonatal gonococcal conjunctivitis is a severe, potentially blinding disease, that can be prevented only if early diagnosis and adequate therapy is available.[2,4,12] The abandonment of prophylaxis would without doubt lead to an increase of ophthalmia neonatorum and its sequela, as this inquiry has shown (Tables 2 and 3).

Prophylaxis against gonococcal conjunctivitis could be terminated if a sufficient, cost-effective screening system for pregnant women (until term) were available. However, there is no such system. Selective application of diagnostic procedures is not an alternative or solution, since gonoccoccal colonization in pregnancy is usually asymptomatic, the definition of certain risk groups is almost impossible,[16] and furthermore such testing imposes an unacceptable stigma. In addition, selective screening does not solve the problem of neonatal gonococcal disease for those who are not examined.

Routine prophylaxis involves expenses in drugs, organization, and personnel. However, these efforts can be kept low and safety can be maintained by the use of standardized, commercially available disposable applicators.

All prophylactic procedures cause corneal irritation (Table 2) and also pain and discomfort.[17,18] However, there is no well-documented case of permanent or severe damage following adequate silver nitrate application.[19–21] Psychological aspects have been considered by several authors, but there is also no evidence that the degree of "eye openness" of the newborn significantly affects the attention of the mother immediately postpartum. The infant has other behaviors and attributes such as cry, body texture, movement, odor, and nursing behavior that facilitate bonding.[22,23]

Like many preventive methods, neonatal ocular prophylaxis does not provide complete protection, as was seen in the inquiry. This holds true for both gonococcal conjunctivitis and other bacterial infections (Table 4), especially chlamydial disease.[24,25] However, the antimicrobial activity of silver nitrate covers a broad spectrum of causative agents regardless of bacterial resistance and without the other inherent characteristic limitations of antibiotics.[4,9,12,15,18,22,24–27] The exception, *C. trachomatis,* should be of no relevance in the future, since the German Society of Gynecology and Obstetrics and the German Society of Perinatal Medicine decided in 1992 to recommend routine screening and therapy for chlamydial infection as a standard of care in pregnancy.[28] In brief, the guidelines emphasize diagnosis as early as possible in pregnancy by a direct test and treatment by erythromycin ethylsuccinate 500 mg q.i.d. for 10 days after the 14th gestational week; partner therapy is mandatory; Credé's prophylaxis with 1% silver nitrate is also recommended by the guidelines, since eye infections of other etiologies may still occur, and no well-studied alternative exists.

The enforcement of Credé's prophylaxis in Germany by law was discontinued in 1986. A restoration of the old status is not desirable, since controversial human rights and legal aspects are of increasing importance. On the other hand, negligence and/or abolition of routine prophylaxis that result in damage to the newborn could probably create another legal issue, the charge of having neglected the current standard of care. This statement accords with the results of this and Viehweg's inquiries and is supported as well by the literature and by several national and international authorities, e.g., the World Health Organization,[10,11,14] the Centers for Disease Control,[13] and a committee of the German Federal Health Department. In Germany it was also supported by at least 80% of ophthalmologists consulted at university hospitals (A.A. Bialasiewicz, personal communication).

Evaluation of the arguments and the literature and the results of this inquiry appear to warrant the following conclusions for Germany, as drawn by the Federal Health Department: gonococcal infection in pregnancy is a rare disease; however, the identification and treatment of infected mothers can not be counted on.[12,16] Therapy of the infected infant appears to involve an unjustifiable risk. Thus neonatal ocular prophylaxis against gonococcal and other infections still appears to represent the standard of care[10,13] and is used in the majority of German hospitals questioned. Silver nitrate 1% aqueous solution is considered a necessary prophylactic measure, safe if properly administered.[4,12,17,19,21,27] A new law is not warranted in Germany for several reasons. The routine administration of such antibiotics as penicillins, aminoglycosides, erythromycin, or tetracyclines cannot be recommended as an alternative since serious untoward reactions (due to poor stability and applicability of the drug, a limited bacteriological spectrum, selection of resistant strains, and possible allergenicity for the newborn) must be taken into account.[9,15,18,25,26] The data on their efficacy are also not superior or convincing enough to justify their routine use in prevention of bacterial ophthalmia neonatorum, particularly if chlamydial infection is detected and treated in pregnancy.[25,26] However, major efforts should be directed toward conducting scientific studies on the replacement of silver nitrate by alternative antiseptic substances (e.g., octenidin or biguanides) in the future.

### REFERENCES

1. Credé CSF: Die Verhütung der Augenentzündung der Neugeborenen. Arch Gynäkol 17:50–53, 1881.
2. Rothenberg R: Ophthalmia neonatorum due to *Neisseria gonorrhoeae:* Prevention and treatment. Sex Transm Dis 6:187–191, 1979.
3. Eder W: Credé-Prophylaxe mit Silbernitrat beim Neugeborenen? Gynäkol Prax 11:627–630, 1987.
4. Dinsmoor MJ: Ophthalmia neonatorum. Contemp Obstet Gynecol 37:112–114, 1992.
5. Laga M, Meheus A, Piot P: Epidemiology and control of gonococcal ophthalmia neonatorum. Bull WHO 67:471–478, 1989.
6. Fransen L, Van den Berghe P, Mertens A, Van Brussel K, Clara R, Piot P: Incidence and bacterial aetiology of neonatal conjunctivitis. Eur J Pediatr 146:152–155, 1987.
7. Huber-Spitzy V, Arocker W, Schmidt C: Ophthalmia neonatorum. Klin Monatsbl Augenheilkd 191:341–343, 1987.
8. Isenberg S: Source of conjunctival bacterial flora at birth and implications for ophthalmia neonatorum prophylaxis. Am J Ophthalmol 106:458–462, 1988.
9. Hedberg K, Ristinen TL, Soler JT, White KE, Hedberg CW, Osterholm MT, MacDonald KL: Outbreak of erythromycin-resistant staphylococcal conjunctivitis in a newborn nursery. Pediatr Infect Dis J 9:268–273, 1990.
10. WHO: Prevention and treatment of conjunctivitis in newborn at the primary level. PBL/84.4 1–23.
11. WHO: Conjunctivitis of the Newborn. Geneva: WHO, 1986.
12. Hammerschlag MR: Neonatal ocular prophylaxis. Pediatr Infect Dis J 7:81–82, 1988.
13. CDC: 1989 Sexually Transmitted Diseases Treatment Guidelines. MMWR 38:1–43, 1989.
14. WHO: Unentbehrliche Arzneimittel. Fünfte Modelliste. Frankfurt/Main, Medico Internat, 1989.
15. Chandler JW: Controversies in ocular prophylaxis of newborns. Arch Ophthalmol 107:814–815, 1989.
16. Brunham RC, Holmes KK, Embree JE: Sexually transmitted diseases in pregnancy. In Holmes KK, Mardh P-A, Sparling PF, Wiesner PJ (eds): Sexually Transmitted Diseases, 2nd ed. New York: McGraw-Hill, pp 771–801, 1989.
17. Nishida H, Risemberg HM: Silver nitrate solution and chemical conjunctivitis. Pediatrics 56:368–373, 1975.
18. Christian JR: Comparison of ocular reactions with the use of silver nitrate and erythromycin ointment in ophthalmia neonatorum prophylaxis. J Pediatr 37:55–60, 1960.
19. Giffin RB: Eye damage in newborns from use of strong silver nitrate solutions. Cal Med 107:178–181, 1967.
20. Hornblass A: Severe silver nitrate ocular damage. NY J Med 1875–1878, October 1976.
21. Schirner G, Schrage NF, Salla S, Teping Ch, Reim M, Burchard W-G, Schwab B: Corneal silver deposits following Credé's prophylaxis. Lens Eye Toxic Res 7:445–457, 1990.
22. Bernstein GA, Davis JP, Katcher ML: Prophylaxis of neonatal conjunctivitis. Clin Pediatr 21:545–550, 1982.
23. Butterfield PM, Emde RN, Svejda MJ: Does the early application of silver nitrate impair maternal attachment? Pediatrics 67:737–738, 1981.
24. Bryant B: Unit dose erythromycin ophthalmic ointment for neonatal ocular prophylaxis. JOGN 13:83–87, 1984.
25. Black-Payne C, Bocchini JA, Cedotal C: Failure of erythromycin ointment for postnatal ocular prophylaxis of chlamydial conjunctivitis. Pediatr Infect Dis J 8:491–498, 1989.
26. Latif A, Mason P, Marowa E, Paraiwa E, Dhamu F, Tambo J, Gwanzura L, Mapeta D, Jongeling G: Management of gonococcal ophthalmia neonatorum with single-dose kanamycin and ocular irrigation with saline. Sex Transm Dis 15:108–109, 1988.
27. Lund RJ, Kibel MA, Knight GJ, Van der Elst C: Prophylaxis against gonococcal ophthalmia neonatorum. S Afr Med J 72:620–622, 1987.
28. Hoyme UB: *Chlamydia trachomatis*–Infektionen in der Schwangerschaft. Perinatal Med 4:53–56, 1992.
